# Feasibility of a Text Messaging–Integrated and Chatbot-Interfaced Self-Management Program for Symptom Control in Patients With Gastrointestinal Cancer Undergoing Chemotherapy: Pilot Mixed Methods Study

**DOI:** 10.2196/46128

**Published:** 2023-11-10

**Authors:** Sameh Gomaa, James Posey, Babar Bashir, Atrayee Basu Mallick, Eleanor Vanderklok, Max Schnoll, Tingting Zhan, Kuang-Yi Wen

**Affiliations:** 1 Department of Medical Oncology Thomas Jefferson University Philadelphia, PA United States; 2 Department of Pharmacology, Physiology, and Cancer Biology Thomas Jefferson University Philadelphia, PA United States

**Keywords:** chemotherapy, gastrointestinal cancer, digital health, text messaging, chatbot, side effect management

## Abstract

**Background:**

Outpatient chemotherapy often leaves patients to grapple with a range of complex side effects at home. Leveraging tailored evidence-based content to monitor and manage these symptoms remains an untapped potential among patients with gastrointestinal (GI) cancer.

**Objective:**

This study aims to bridge the gap in outpatient chemotherapy care by integrating a cutting-edge text messaging system with a chatbot interface. This approach seeks to enable real-time monitoring and proactive management of side effects in patients with GI cancer undergoing intravenous chemotherapy.

**Methods:**

Real-Time Chemotherapy-Associated Side Effects Monitoring Supportive System (RT-CAMSS) was developed iteratively, incorporating patient-centered inputs and evidence-based information. It synthesizes chemotherapy knowledge, self-care symptom management skills, emotional support, and healthy lifestyle recommendations. In a single-arm 2-month pilot study, patients with GI cancer undergoing chemotherapy received tailored intervention messages thrice a week and a weekly Patient-Reported Outcomes Version of the Common Terminology Criteria for Adverse Events–based symptom assessment via a chatbot interface. Baseline and postintervention patient surveys and interviews were conducted.

**Results:**

Out of 45 eligible patients, 34 were enrolled (76% consent rate). The mean age was 61 (SD 12) years, with 19 (56%) being females and 21 (62%) non-Hispanic White. The most common cancer type was pancreatic (n=18, 53%), followed by colon (n=12, 35%) and stomach (n=4, 12%). In total, 27 (79% retention rate) participants completed the postintervention follow-up. In total, 20 patients texted back at least once to seek additional information, with the keyword “chemo” or “support” texted the most. Among those who used the chatbot system checker, fatigue emerged as the most frequently reported symptom (n=15), followed by neuropathy (n=7). Adjusted for multiple comparisons, patients engaging with the platform exhibited significantly improved Patient Activation Measure (3.70, 95% CI –6.919 to –0.499; *P*=.02). Postintervention interviews and satisfaction surveys revealed that participants found the intervention was user-friendly and were provided with valuable information.

**Conclusions:**

Capitalizing on mobile technology communication holds tremendous scalability for enhancing health care services. This study presents initial evidence of the engagement and acceptability of RT-CAMSS, warranting further evaluation in a controlled clinical trial setting.

## Introduction

Cancer treatments have increasingly transitioned to ambulatory settings, where chemotherapy is administered on an outpatient basis. This shift places a significant responsibility on patients to independently manage a spectrum of intricate and varied side effects, often without immediate clinical assistance. The array of symptoms experienced during chemotherapy, including intense fatigue, persistent nausea, and acute pain, can be both frequent and severe. Detecting emerging, intensifying, and unforeseen side effects hinges on patients’ capacity to differentiate between anticipated and alarming symptoms and promptly access suitable medical attention. Unfortunately, many patients are inadequately equipped for this task, therefore frequently deferring seeking help [1,2]. Such delays can markedly compromise their quality of life and adherence to chemotherapy regimens. Research indicates that clinicians routinely underestimate the severity of their patients’ symptoms compared to patients’ self-reported experiences, resulting in suboptimal health outcomes and potentially inadequate treatment [3]. Notably, the prevalence of gastrointestinal (GI) cancers is striking, with millions of cases being diagnosed worldwide annually [4]. Within this demographic, the challenges are exacerbated by the complex interplay between the gastrointestinal system and chemotherapy agents. GI cancers carry adverse clinical implications such as reduced chemotherapy tolerance, heightened incidence of toxicities, surgical complications, extended hospital stays, increased infection risks, and diminished overall survival [5,6]. The burden of chemotherapy in the context of GI cancers is substantial, given the need for enhanced monitoring and support to mitigate potential complications. This combination of high prevalence, the intricate burden of chemotherapy, and the unique challenges faced by patients with GI cancer underscores the pertinence of focusing on this population. Furthermore, the insights gleaned from studying GI cancer management possess the potential for broader applicability beyond this specific cancer type, offering valuable guidance for addressing challenges in diverse cancer contexts.

Emerging studies have highlighted the pivotal role of remote patient monitoring in ambulatory cancer care, particularly through the implementation of electronic patient-reported outcomes (ePROs) to manage symptoms effectively. Notably, numerous ePRO systems have demonstrated their capacity to enhance patient satisfaction, foster patient-clinician communication, improve health-related quality of life, bolster overall survival rates, and curtail the usage of acute health services [7-9]. However, it is essential to recognize that the evaluation of ePRO systems’ impact within the realm of patients with GI cancer remains limited, warranting further investigation. In tandem with these advancements, text messaging–based interventions have garnered attention as valuable tools for managing cancer treatment, particularly during chemotherapy. These interventions, although not directly communicated by health care providers, have exhibited the potential to not only monitor symptoms but also to furnish information on prevention, strategies for managing side effects, and emotional support [10,11]. In addition, chatbots, automated conversational agents, have emerged as an innovative solution, particularly noteworthy for their perceived accessibility by older patients with cancer [12]. By using a structured set of questions akin to the patient encounter review of systems conducted by a nurse, chatbots offer a streamlined and engaging approach. Encouragingly, pilot studies have demonstrated the feasibility and acceptability of chatbot apps to monitor symptoms among patients with older cancer undergoing chemotherapy at home [12].

In line with these developments, we propose a hybrid approach that amalgamates the unique strengths of both text messaging and chatbot platforms. This hybrid model presents an intuitive and engaging platform tailored to the needs of patients with GI undergoing chemotherapy. By seamlessly integrating text messaging–based interventions and chatbot capabilities, this hybrid version offers patients a multifaceted solution. It empowers them to access proactive supportive information, evaluate a spectrum of symptoms, and receive real-time tailored self-care advice. Through this innovative synergy of technology, we anticipate the creation of a user-friendly and efficient system that optimizes the care experience for patients with GI cancer receiving chemotherapy, ultimately contributing to improved outcomes and enhanced patient well-being.

## Methods

### Study Aim

The aim of the pilot study was to develop and implement a text messaging–based system, Real-time Chemotherapy-Associated Side Effects Monitoring Supportive System (RT-CAMSS), for patients with GI receiving chemotherapy. In RT-CAMSS, participants were proactively sent 3 patient education and supportive text messages per week and prompted weekly by a text to assess symptoms via a semistructured chatbot interface. Patients were encouraged to text back specific keywords to the system for additional corresponding information and coping strategies and were provided with evidence-based real-time recommendations to support their self-management tailored to their symptom assessment responses.

### Ethical Considerations

The study was granted approval by Thomas Jefferson University’s institutional review board (IRB# 20G.090) and the Sidney Kimmel Cancer Center’s ethics committee. Prior to participation in the study, written informed consent was acquired from all participants. Comprehensive steps were taken to ensure the anonymity of all collected data, including interview transcripts, survey responses, and data obtained through text messages or the chatbot interface. The text messaging–delivered app operates within the framework of Health Insurance Portability and Accountability Act security compliance, ensuring that patient data remain protected and confidential. For text message exchanges, we used end-to-end encryption, ensuring that messages remained private between patients and the system. Similarly, chatbot interactions are designed to be secure, with patient data being deidentified to prevent any traceability. These measures collectively uphold the privacy and security of patient interactions within the RT-CAMSS System, aligning with the ethical standards and regulations governing the protection of patient information. The study strictly adhered to national and international regulations pertaining to the safeguarding of personal information, privacy, and human rights, as necessitated by the use of digital technology.

### Study Design

The study design encompassed a mixed methods approach consisting of two primary phases: (1) phase 1 dedicated to system development and (2) phase 2 focused on executing a pilot intervention spanning 2 chemotherapy cycles. Phase 2 represented a single-arm pilot study aimed at quantifying the feasibility and acceptability of the RT-CAMSS intervention. This phase specifically evaluated the following aspects: (1) enrollment and retention rates, (2) intervention usage across a 2-month duration, (3) patient acceptability and satisfaction regarding the intervention, and (4) changes in quality of life and related metrics from baseline to the 2-month follow-up assessment.

### Phase 1: Development Phase

#### Overview

The aim of phase 1 is to develop and refine the RT-CAMSS intervention through a systematic process that draws upon evidence-based information and patient-centered interview data. This phase serves as the foundational step in crafting an intervention that is both informed by scientific knowledge and tailored to meet the specific needs and preferences of the patient population.

In phase 1, we undertook a comprehensive review of the literature and consulted evidence-based guidelines concerning optimal practices in symptom management. This step was pivotal in shaping the development of our self-care message tips and in establishing the thresholds for symptom severity alerts. Our search was tailored to identify relevant studies, guidelines, and resources focused on optimal symptom management practices. We delved into databases such as PubMed, Cochrane Library, and relevant oncology-specific repositories, using keywords and Medical Subject Headings terms. Our inclusion criteria encompassed studies published within a defined time frame, focusing on patients with GI cancer undergoing chemotherapy. Articles discussing symptom management strategies [13], treatment guidelines, and patient perspectives were prioritized. Data extraction involved meticulous scrutiny of selected studies, wherein we gathered insights on symptom prevalence, severity thresholds, and evidence-based coping strategies. Drawing from established studies using tools like the National Cancer Institute (NCI) Patient-Reported Outcomes Version of the Common Terminology Criteria for Adverse Events [14-16], we carefully adopted thresholds and guidelines that are widely acknowledged in the field. The formulation of the alert algorithm was a crucial aspect of this development phase. We designed the algorithm to identify reported symptoms that necessitate patient-clinician communication or immediate medical attention. Simultaneously, we incorporated coping strategies to assist patients in managing these symptoms effectively. In line with a patient-centered approach, we conducted insightful interviews with patients with GI cancer who had prior chemotherapy experiences. These interviews were instrumental in fine-tuning the selection of symptoms and curating the content of the self-care messages. This approach not only enriched the relevance of our intervention but also ensured that the insights directly reflected the unique needs and concerns of the patient population.

Seven participants were involved in the qualitative interview, including 4 female participants, with a mean age of 59.8 (SD 12.1) years. Among them, 4 were married, and the average time since diagnosis was 22 (SD 13.2) months. All participants underwent both surgery and chemotherapy. The interview revealed 3 primary themes described below. Corresponding patient quotes for each theme are listed in Textbox 1.

**Patient interview quote samples for each theme.**

**Needs for symptom management**

- *So what truly bothered me the most about chemotherapy where my fingers, hands and feet.*
- *I just can't put my feet while I went to a beach. I couldn't put my feet in the water, just cause it freezes off.*
- *my taste buds, um, were off significantly. My food, I just, all the food I ate was bad. It tasted terrible.*
- *I know for sure that my memory is not as good as it was. Chemo brain as they call it or whatever.*
- *Everything. Everything. I couldn't do anything. Cause I was so weak from the chemo and radiation.*

**Needs for emotional management**

- *My emotional state was, I didn't feel very safe. I felt very scared. I felt a lot of mistrust. I didn't like the environment.*
- *it wasn't all physical side effects. It was emotional side effects too. It was fear. It was mental side effects.*
- *It is an emotional roller coaster. Um, some days you just so bad, you know, you have, um, horrible thoughts.*

**Needs for information acquisition**

- *I knew nothing of what to expect or, how to what I was going to feel or anything.*
- *I think I knew most of the stuff. I guess just the side effects of, the short term and long term side effects of it. I wasn't too sure about how functional I would be the day of afterwards and stuff like that.*
- *I learned afterwards, that physical, some kind of physical exercise of the body is very important to do during chemotherapy.*
- *My advice would be to, somebody tells, you know, give them all the information, what side effects are possible. Not that you're going to get them all because everybody is different, but this is what you need to plan for just in case.*

#### Needs for Symptom Management

During chemotherapy, participants faced a range of disruptive symptoms that affected their daily lives. The identification of specific side effects and knowing when to seek medical attention were challenging. Neuropathy emerged as a prevalent and troublesome symptom, alongside fatigue, cognitive issues, and gastrointestinal disturbances such as nausea, vomiting, and diarrhea. Some participants vividly described the impact of neuropathy on their ability to perform simple tasks, while others highlighted the loss of taste during treatment.

#### Needs for Emotional Management

Patients shared their emotional struggles, expressing feelings of being overwhelmed, scared, and fearful. Emotional distress was noted as an integral part of the cancer journey alongside physical side effects. Participants described the emotional roller coaster they experienced, including days marked by negative thoughts and profound fear. The environment and uncertainty contributed to mistrust and discomfort for some participants.

#### Needs for Information Acquisition

Patients highlighted the importance of information and preparation before and during treatment. Many expressed feeling uninformed about what to expect during their treatment journey, both in terms of physical experiences and functionality. Some mentioned the necessity for exercise guidance during chemotherapy, indicating a need for comprehensive resources. Additionally, participants stressed the importance of receiving information about possible side effects to better plan for their experiences.

The insights garnered from these interviews underscore the multifaceted needs of individuals undergoing chemotherapy for GI cancers. These findings emphasize the significance of addressing not only physical symptoms but also emotional well-being and the provision of comprehensive information to empower patients throughout their chemotherapy process. Understanding these aspects helped us to develop an RT-CAMSS system that addresses the needs of patients with GI cancer undergoing chemotherapy.

### Phase 2: Pilot Intervention

#### Overview

The aim of phase 2 is to conduct a 2-month pilot test of the RT-CAMSS intervention within the patient population having GI cancer. This phase seeks to assess the intervention’s feasibility, as indicated by accrual and attrition rates. Additionally, it aims to gauge the intervention’s acceptability by measuring patient satisfaction ratings and conducting postintervention interviews. By engaging participants in the intervention, we aim to evaluate its practical implementation, user acceptance, and its potential to enhance quality of life, patient activation, and symptom distress management. The insights gathered from this pilot phase will guide the refinement of the intervention, ensuring its relevance and effectiveness for patients with GI cancer.

#### RT-CAMSS System

##### Overview

Based on the insights gleaned from our phase 1 interviews and a thorough review of evidence-based resources, the RT-CAMSS intervention was thoughtfully crafted with 3 essential functions.

##### Proactive Interactive Text Messages

Participants received proactive and interactive text messages 3 times a week. These messages covered a spectrum of vital topics, including chemotherapy-related knowledge, strategies for preventing and managing side effects, recommendations for self-care and lifestyle behavioral changes, emotional support, and guidance on effective communication with health care providers and family members. Encouragingly, patients were prompted to engage further by texting predefined topic keywords, thereby requesting additional information tailored to their specific needs.

##### Chatbot Symptom Monitoring

A chatbot interface was seamlessly integrated into the intervention, using text messages to regularly monitor participants’ symptoms. This feature allowed participants to assess their symptoms at their convenience, providing them with a user-friendly and accessible means of reporting their experiences.

##### Immediate Self-Care Feedback

An integral aspect of the intervention was the provision of immediate feedback based on the symptom assessments collected via the chatbot. This feedback offered participants valuable guidance on self-care actions to be taken in response to their reported symptoms. This timely and tailored support aimed to empower patients with actionable steps to manage their symptoms effectively.

**Figure 1:**
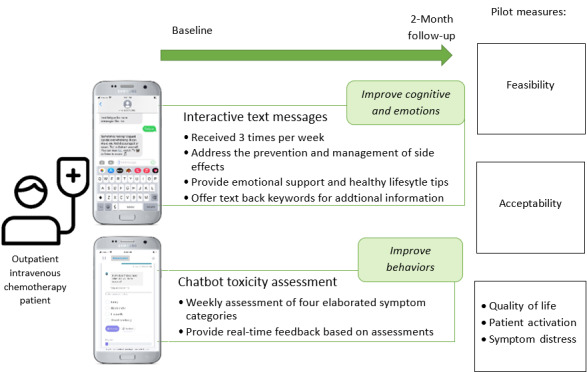
RT-CAMSS intervention and study design. RT-CAMSS: Real-Time Chemotherapy-Associated Side Effects Monitoring Supportive System.

By encompassing these 3 interconnected functions, the RT-CAMSS intervention was holistically designed to address the diverse needs of patients with GI cancer undergoing chemotherapy. It aimed not only to provide information but also to actively engage patients, monitor their well-being, and offer real-time guidance for symptom management and self-care. This multifaceted approach sought to enhance the overall quality of care and support provided to patients during their challenging treatment journey. Figure 1 shows the design of the intervention and study.

The chatbot app, pivotal to our study, operated as a semiautomatized messaging system optimized for smartphones. Our decision to use a text messaging–delivered app over a native app was primarily due to accessibility and cost factors. The symptom checker tool is electronically connected and therefore not stored on a user’s device. Our chatbot approach centered on structured questions and tailored responses. By adopting this method, the chatbot engaged users through predetermined questions designed to gather specific information. Subsequently, the chatbot provided responses that were meticulously tailored to the input received, thereby delivering personalized information and guidance based on users’ interactions.

The creation of the chatbot involved a collaborative effort encompassing expertise in oncology, technology, and data security. This ensured that the chatbot’s functionalities aligned closely with medical guidelines while also prioritizing ease of use and accuracy in information delivery. Through its integration into a smartphone-based messaging system, the chatbot facilitated convenient and accessible communication, allowing patients to engage in meaningful exchanges with the technology at their own pace and convenience. Furthermore, our chatbot was carefully crafted to meet rigorous health data security standards, guaranteeing the safety of sensitive patient information. Its adherence to Health Insurance Portability and Accountability Act regulations underscored our unwavering commitment to upholding the confidentiality and privacy of patients’ data, thereby cultivating an environment of trust within the platform.

**Figure 2:**
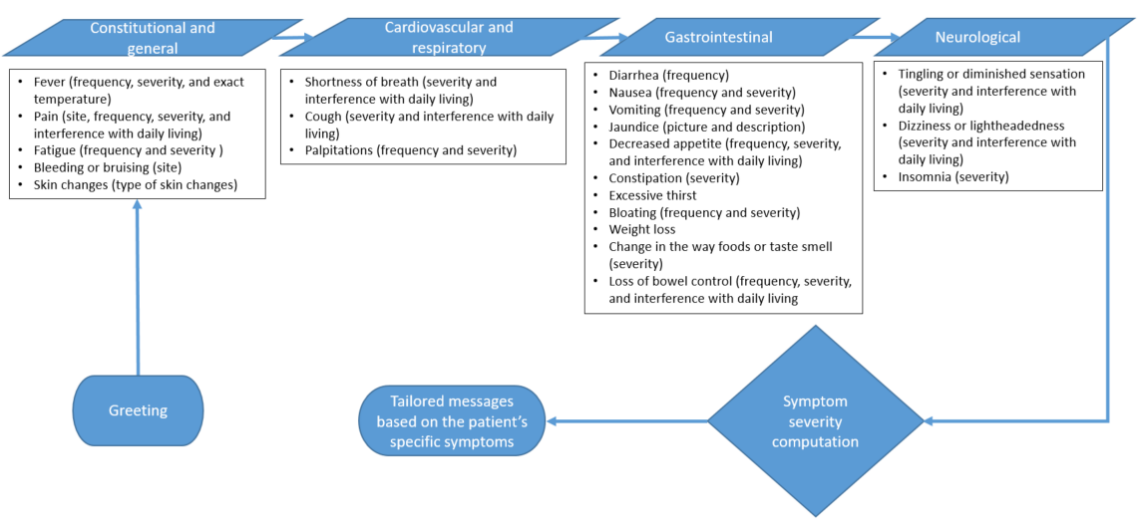
Chatbot symptom checker assessment flow.

**Figure 3:**
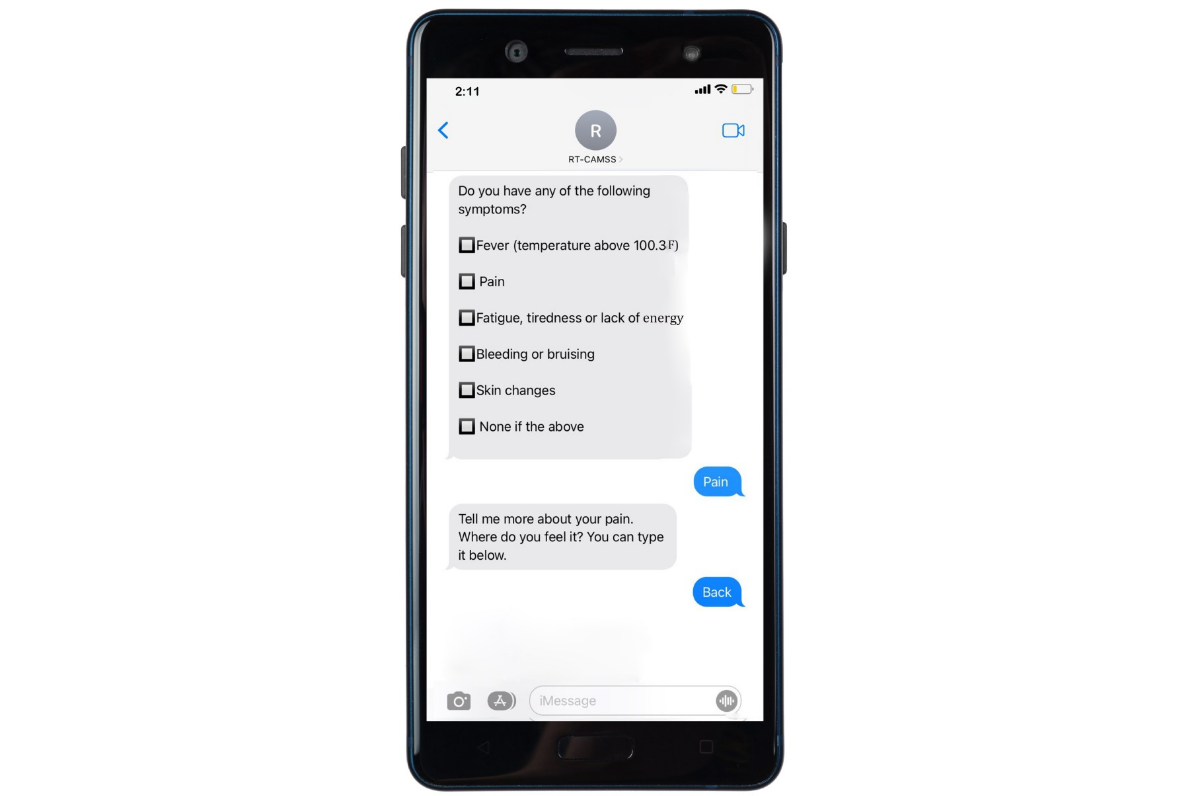
Chatbot interaction sample.

**Figure 4:**
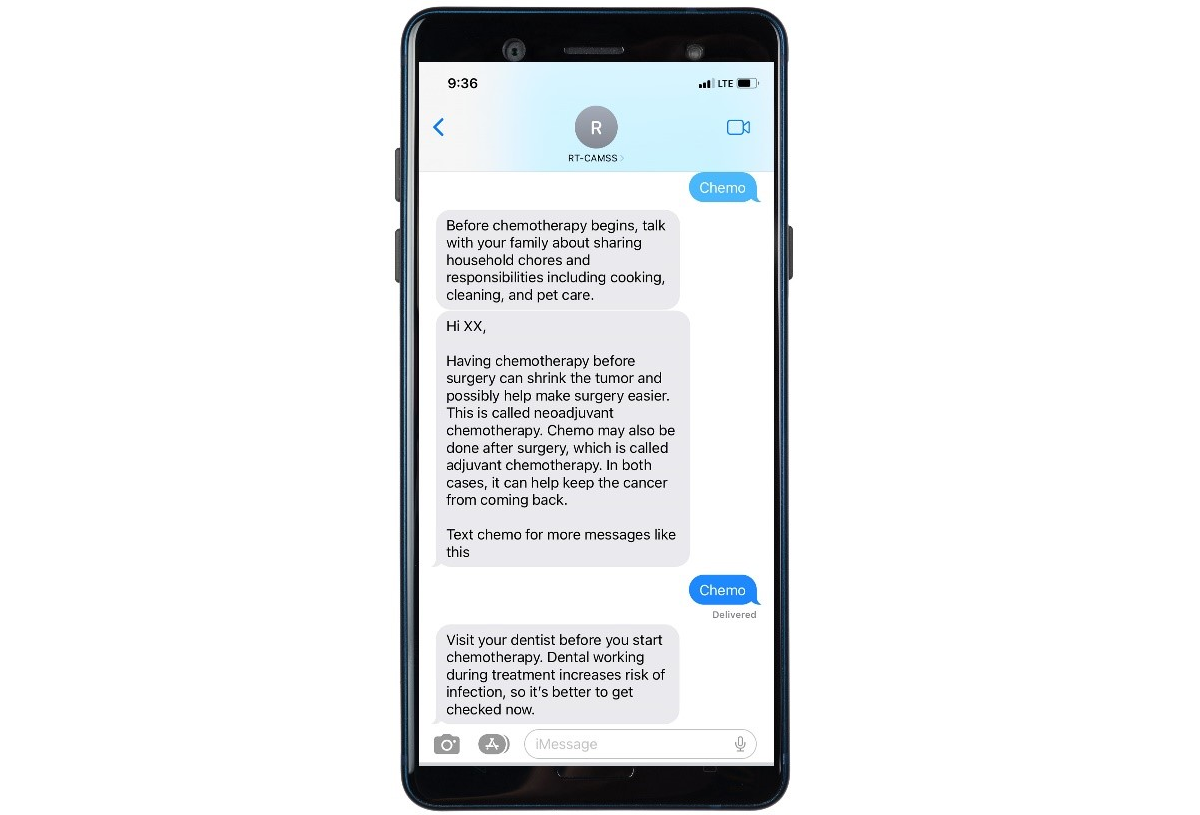
Sample text message interaction.

The symptom domains, as depicted in Figure 2, encompass constitutional and general, cardiovascular and respiratory, GI, and neurological categories. These domains emerged through the consolidation of prevalent adverse events linked to individual chemotherapeutic agents used in treating gastrointestinal malignancies. Notably, these agents encompass platinum-based compounds, antimetabolites (5-fluorouracil and Capecitabine), anthracycline, mitotic inhibitors (Docetaxel and Paclitaxel), and topoisomerase inhibitors (Irinotecan). The assessment of severity and frequency was adapted from the PRO CTCAE (Patient-Reported Outcomes version of the Common Terminology Criteria for Adverse Events) framework. A concise set of sequential questions was transmitted to patients weekly to systematically monitor the prevalence and severity of the identified symptoms. The design of the question flow mirrored the review of systems section typically used during clinical encounters. The overarching objective was to effectively screen patients for common side effects linked to their chemotherapy treatment, both acute and latent in nature. Subsequent to this assessment, the chatbot engaged patients in personalized education and symptom management recommendations, enhancing the interactive nature of the intervention. The algorithmic structure aligned with CTCAE’s (Common Terminology Criteria for Adverse Events’) established grading definitions: (1) for grade 1 or grade 2 symptoms, tailored self-care advice and access to evidence-based resources were provided; (2) in the case of grade 3 reported symptoms, participants were encouraged to promptly reach out to their health care team; and (3) for grade 4 symptoms, participants were advised to seek immediate assistance from the nearest emergency room. Importantly, participants were reminded that the information gathered remained confidential and was not shared with their health care providers. It was also underscored that the advice dispensed by the intervention should not be considered a substitute for medical guidance or appointments. Examples illustrating the interactions with the chatbot symptom checker are visually presented in Figure 3. An illustrative sample of an interactive text message is showcased in Figure 4, offering a tangible depiction of the user experience within the intervention.

### Patient Population and Recruitment

Eligible patient participants, aged 18 years or older, were required to have a diagnosis of malignant GI cancer at any stage, should be undergoing or planning intravenous chemotherapy treatment at the studied cancer center, should possess English language proficiency for both speaking and reading, and have access to a smartphone with text messaging capabilities. Patients with documented cognitive impairment or psychiatric depression were excluded from participation.

During their infusion appointments, potentially eligible patients were approached and given the opportunity to seek clarifications or opt out if not comfortable. Once written consent or e-consent was secured, participants completed a baseline survey and were requested to engage with the intervention for a duration of 2 months. Research staff maintained biweekly contact through in-person meetings or telephone calls to ensure participant progress and address any queries.

### Measures

Clinical characteristics encompassing diagnosis, stage, and treatment regimen were extracted from participants’ medical records. Preintervention and postintervention assessments involved the completion of various instruments. Functional Assessment of Cancer Therapy has been validated for use in oncology clinical trials and practice for measuring quality of life [17]. The Memorial Symptom Assessment Scale has been adapted to assess the symptom experience and burden of patients with cancer as a valid and reliable instrument [18]. The Patient Activation Measure (PAM) has been validated in measuring perceived capacity in managing own health including cancer treatments [19]. The Multidimensional Scale of Perceived Social Support has been used in behavioral trials for measuring perceived social support among patients with cancer [20]. The Cancer Treatment Survey instrument was developed and validated to assess the preparation for chemotherapy and radiotherapy in patients with cancer [21]. To evaluate intervention satisfaction, we used a succinct survey created by our research team. This survey, administered post intervention, was designed to assess the usability and acceptability of the intervention. Usability was gauged by evaluating the clarity of messages and the appropriateness of timing and frequency (2 items). The survey also assessed the program’s helpfulness in terms of aiding patients in coping with and understanding the side effects of chemotherapy, enhancing their knowledge about the benefits and risks of chemotherapy, and facilitating communication with health care providers (3 items). All satisfaction questions were measured on a 1-5–point scale.

To collect survey responses, participants were provided with a survey link via email or text through the Research Electronic Data Capture (REDCap; Vanderbilt University) web-based platform. Alternatively, paper questionnaires were offered during clinical visits. In recognition of their time and contribution, participants received gift cards valued at US $20, US $30, and US $40 for successive time points.

### Postintervention Interviews

Participants were extended an invitation to engage in semistructured phone interviews, which are designed to glean insights into their experiences and feedback regarding the RT-CAMSS intervention. The interviews delved into various aspects, encompassing the ease of use, the pertinence of self-care messages, encounters with symptoms not covered by the chatbot’s toxicity assessment, and suggestions for enhancing the chemotherapy experience.

### Data Analysis

The statistical analysis was executed using R (R Foundation for Statistical Computing). Basic demographic and medical characteristics are summarized by descriptive statistics. The qualitative data analysis involved systematically identifying themes and categories from the interview transcriptions using NVivo software (QSR International). Initially, a codebook was created based on topical codes from the interviews, which are enriched with interpretive codes during ongoing analysis. This process ensured organized data grouping into relevant themes. To enhance trustworthiness, intercoder reliability was established through independent coding by multiple researchers, followed by consensus discussions. Member checking involved sharing findings with participants to validate accuracy. This iterative process of coding, discussion, and validation ensured credible qualitative findings.

Engagement with the intervention, including the frequency of patient-initiated text responses for additional tips and responses to the chatbot’s toxicity assessment, is outlined using descriptive statistics Summary statistics of Functional Assessment of Cancer Therapy, Multidimensional Scale of Perceived Social Support, PAM, Memorial Symptom Assessment Scale, and Cancer Treatment Survey scores at baseline and the 2-month follow-up are computed. Paired *t* tests were performed to compare 2-month scores with baseline scores for each measure, and the results with 95% CIs are presented. Additionally, an exploration of whether patient engagement impacted their perceived activation was conducted. Patients were categorized based on whether they had texted back for more information or responded to the toxicity survey and whether they had used the Chatbot. Analysis was performed in R, assessing differences in averages using paired *t* tests.

## Results

### Sample Characteristics

Out of the 50 individuals approached for participation in the pilot study, 34 provided their consent. The sample consisted of 19 women, with an average age of 61 (SD 12) years. The majority were married or had a partner (n=21, 62%), possessed a college degree or higher (n=28, 79%), and had an income exceeding US $60,000 (n=20, 59%). Among the participants, 18 (53%) were diagnosed with pancreatic cancer, 27 (79%) had a cancer stage beyond stage II, and 23 (68%) were within 3 months of their initial chemotherapy cycle at enrollment. Further details regarding the characteristics can be found in Table 1.

**Table 1:** Sociodemographic and medical characteristics of study participants.

Characteristics		Values (N=34)
Age (years), mean (SD)		60.97 (12.00)
**Race, n (%)**		
	Caucasian	21 (62)
	Minority	13 (38)
**Sex, n (%)**		
	Male	15 (44)
	Female	19 (56)
**Marital status, n (%)**		
	Married or with a partner	21 (62)
**Education level, n (%)**		
	High school	6 (18)
	College graduate	16 (47)
	Post graduate school	11 (32)
	Unknown	1 (3)
**Income level, n (%) (US $)**		
	<$30,000	6 (15)
	$30,000-$60,000	8 (24)
	>$60,000	20 (59)
	Unknown	1 (3)
**Cancer type, n (%)**		
	Pancreatic	18 (53)
	Colorectal	12 (35)
	Gastric or esophageal	4 (12)
**Cancer stage, n (%)**		
	I-II	7 (21)
	III-IV	27 (79)
**Duration on chemotherapy at the time of enrollment (months), n (%)**		
	<3	23 (68)
	3-10	8 (24)
	>10	3 (9)

### Feasibility

Feasibility was assessed based on study accrual and attrition rates. A feasibility criterion of an accrual rate exceeding 65% and attrition below 30% was applied. Out of 50 eligible patients approached, 34 consented and enrolled (68% consenting rate). Post intervention, 27 (20.6% attrition rate) participants successfully completed the follow-up. These outcomes affirm the feasibility of the intervention.

### Acceptability

Acceptability was measured through a satisfaction survey and patient interview data. The results of the assessment indicate high levels of participant satisfaction with the intervention. The usability domain yielded a mean score of 4.47 (SD 0.45), while the helpfulness domain yielded a mean score of 3.73 (SD 0.85), both measured on a 1-5–point scale. These scores underscore the favorable reception of the intervention among participants, reflecting their positive perception of the usability and beneficial aspects of the program.

### Postintervention Interviews

Through 9 completed interviews, significant emotional benefits and valuable self-regulatory skills emerged from using the intervention. Patients highlighted how the system assisted them in understanding and coping with side effects, leading to a sense of empowerment. Encouraging messages provided solace amidst the physical challenges, helping patients maintain positivity and engagement. In particular, during the isolating times of the COVID-19 pandemic, the program served as a source of connection and comfort. Patients also expressed appreciation for the intuitive nature of the program. The easy access to information linked to their symptoms enhanced their understanding and decision-making. Importantly, the program was seen to foster better communication with the care team, making patients more open to discussing their condition. The adaptability of the information was emphasized, with patients valuing succinct tips that resonated with their specific experiences. This personalized approach allowed them to absorb and apply advice according to their priorities. In addition, patients offered insights for program enhancement, suggesting connections to other cancer survivors for peer support. They also recommended addressing the needs of patients who are terminally ill, demonstrating a holistic approach to the program’s development. The quotes corresponding to these findings have been extracted from the interviews and can be found in Textbox 2.

**Postintervention participant interview quotes.**

**Helpfulness in coping with side effects**

- *I was having some of the symptoms, but I didn't know that it was related to chemo when I read the messages everything came together. I actually enjoyed it.*
- *It told me a lot about my loss of appetite and this was really helpful, it helped in how to deal with issues and side effects.*

**Helpfulness in coping with emotions**

- *The encouraging messages, they help you take your mind off of the physical aspect.*
- *It helped. I mean, I just stayed positive and stuff and read some of the little stuff y'all used to give.*

**Helpfulness in coping with COVID-19**

- *During COVID we were isolated and it helped somewhat relieve.*

**Relatable**

- *I wasn't really able to talk with anybody about my condition and within the text message, it was like relating, I mean, relating to my condition and that I can be able to talk.*

**Easy to use**

- *I like the additional support and information that I could find on the symptoms that I was having. Everything was linked to the message. If I needed the information, you just hit the link.*

### Text Messaging Interaction

Our content comprises unprompted messages, accompanied by topic keywords that patients can use to access additional tips or coping strategies if needed. Over the 2-month intervention period, 20 (59%) patients engaged by texting back at least once to seek additional information. The range of interactions varied from 1 message to 33 messages, with an average of 15 (SD 4) messages during this period. Notably, the most frequently used keyword was “chemo,” which was inquired about 30 times. Following closely, the second most inquired keyword was “support,” requested a total of 20 times (see Table 2).

**Table 2:** System engagement data.

RT-CAMSS^a^ components	Most used keywords and reported symptoms	Mean (frequency range)
Texting back (20 patients used)	“Chemo” (N=30) “Support” (N=20)	15 (1-33)
Chatbot symptom checker (27 patients used)	Grade 2 Fatigue (n=7) Neuropathy (n=7) Grade 3 Fatigue (n=8) Altered taste (n=5) Decreased appetite (n=5) Grade 4 Constipation (n=2)	4.5 (1-16)

^a^RT-CAMSS: Real-Time Chemotherapy-Associated Side Effects Monitoring Supportive System.

### Reported Symptoms via Chatbot

During the 2-month intervention, 27 (79%) patients used the chatbot system checker at least once. Notably, grade 2 symptoms were predominantly fatigue (n=7) and neuropathy (n=7). For grade 3 symptoms, fatigue (n=8) emerged as the most common, followed by altered taste (n=5) and decreased appetite (n=5). Notably, constipation was the most frequently reported grade 4 symptom (n=2) among 4 patients who reported such symptoms. Collectively, fatigue was the most frequently reported symptom across all grades (n=15), followed by neuropathy (n=7) and altered taste (n=5; see Table 2).

### Change in Psychosocial Measures From Baseline to 2-Month Follow-Up

Comprehensive descriptive data and comparisons between the baseline and 2-month follow-up time points concerning quality of life, symptom distress, and psychosocial measures are presented in Table 3. Using paired sample *t* tests along with 95% CIs, the analysis revealed no statistically significant alterations across all study measures from baseline to the postintervention assessment.

**Table 3:** Changes in measures from baseline to follow-up.

Measure (range)	Baseline (n=34), mean (SD)	2-Month follow-up (n=27), mean (SD)	2-Month change: paired difference (95% CI)	*P* value (paired *t* test)
FACT_PWB^a^ (0-28)	17.88 (5.24)	17.33 (5.55)	0.296 (–1.90 to 2.49)	.78
FACT_SWB^b^ (0-28)	19.44 (4.68)	18.85 (4.87)	–1.000 (–2.48 to 0.48)	.17
FACT_EWB^c^ (0-24)	18.26 (3.37)	17.74 (3.89)	–0.741 (–2.10 to 0.62)	.27
FACT_FWB^d^ (0-28)	16.00 (5.58)	16.52 (5.35)	–2.296 (–4.25 to –0.33)	.24
MSAS^e^ (0-116)	28.41 (13.50)	32.67 (17.74)	4.185 (–2.30 to 10.67)	.19
PAM^f^ (13-52)	39.82 (6.30)	40.28 (5.70)	–0.208 (–1.99 to 1.57)	.81
MSPSS^g^ (12-84)	74.35 (12.07)	73.19 (13.67)	–2.095 (–4.52 to 0.33)	.09
CATS^h^ Psychology subscale	4.16 (6.77)	3.00 (1.09)	–1.596 (–4.62 to 1.43)	.28
CATS_Sensory subscale	2.81 (0.84)	2.81 (0.90)	–0.047 (–0.29 to 0.19)	.69

^a^FACT PWB: Functional Assessment of Cancer Therapy—Physical Well-Being.

^b^FACT SWB: Functional Assessment of Cancer Therapy—Social Well-Being.

^c^FACT EWB: Functional Assessment of Cancer Therapy—Emotional Well-Being.

^d^FACT FWB: Functional Assessment of Cancer Therapy—Functional Well-Being.

^e^MSAS: Memorial Symptom Assessment Scale.

^f^PAM: Patient Activation Measure.

^g^MSPSS: Multidimensional Scale of Perceived Social Support.

^h^CATS: Cancer Treatment Survey.

### Active User Subgroup Analysis in Relation to Patient Activation

A comparison analysis was conducted between text-back users and nonusers on the PAM, as well as between chatbot toxicity users and nonusers from baseline to the 2-month follow-up. The results indicated that patients who engaged in texting back for additional information through the system displayed a noteworthy improvement in PAM with a value of 3.70 (95% CI –6.919 to –0.499), denoted by a *P* value of .03, as outlined in Table 4. In contrast, there was no statistically significant change observed over time in patient activation between chatbot toxicity users and nonusers.

**Table 4:** Change in patient activation score between active users versus nonusers.

Group comparison	Paired difference (95% CI)	*P* value (paired *t* test)
Text back user vs non user	3.701 (–6.92 to –0.50)	.02
Chatbot toxicity user vs nonuser	1.98 (–5.56 to 1.61)	.27

## Discussion

### Principal Findings

This study underscores the feasibility and acceptability of an interactive text messaging–based symptom monitoring and patient education program tailored for individuals with GI cancer undergoing intravenous chemotherapy. Given that GI cancers contribute significantly to global cancer incidence [4], with millions of new cases each year, our initiative seeks to bridge the care gap that often arises during chemotherapy. This gap becomes particularly pronounced in light of disruptions like those posed by the COVID-19 pandemic. Our system, RT-CAMSS, addresses not only symptom tracking but also proactive patient education and support needs, a vital component in managing treatment-related adverse events.

During the program’s development, insights from structured phase 1 interviews played a pivotal role. By aligning the system’s focus with patient needs—ranging from symptom management to emotional support and information acquisition—we crafted RT-CAMSS to be accessible and beneficial. The system’s dual text messaging and chatbot interfaces cater to patient interactions, fostering engagement, and empowering patients to manage their well-being more effectively.

Participants attested that their engagement with RT-CAMSS fostered heightened feelings of reassurance and security, attributed to the heightened awareness of their symptoms and their changes over time. These sentiments were reinforced by the tangible outcomes of the system, where feedback from participants indicated their active implementation of the advice they received. Encouragingly, participants identified the inherent potential of RT-CAMSS to evolve, both in terms of its functional capabilities and its integration into the broader health care landscape. Moreover, while the intrinsic value of such systems is evident, their ultimate impact warrants evaluation through the lens of potential cost savings in patient outcomes. This consideration is particularly significant in the context of mitigating health care usage, such as minimizing emergency department visits or hospitalizations, thereby advancing both patient well-being and health care resource optimization.

Patient engagement and proactive behavior in seeking information and strategies to manage the challenges of chemotherapy were evident through their interaction with the intervention. Notably, the most frequently used keyword in text messaging was “chemo,” closely followed by “support.” A notable finding was that 27 participants actively used the chatbot system checker during the 2-month intervention period. Further, grade 2 symptoms, including fatigue and neuropathy, were the most frequently reported. Grade 3 symptoms also showcased fatigue as a predominant concern, along with notable reports of altered taste and decreased appetite. This pattern aligns with established literature, emphasizing that fatigue and neuropathy are significant challenges faced by patients with GI undergoing chemotherapy [22]. These findings underscore the pertinence of our intervention in addressing these prominent issues and catering to patients’ real-time needs.

A significant finding emerged as participants who actively engaged with the system reported notable improvements in their scores on the PAM. This shift indicates an increase in their confidence, knowledge, and proactive participation in managing their health and symptoms. The use of RT-CAMSS facilitated patients in navigating the intricacies of chemotherapy side effects, showcasing the system’s potential to enhance patients’ self-efficacy and self-regulation in symptom management. The act of proactively seeking additional information demonstrates their growing sense of ownership over their well-being. By providing tailored information and support, the program nurtures patients’ abilities to take charge of their health, fostering a sense of empowerment and activation.

The RT-CAMSS intervention has demonstrated robust feasibility and acceptability across various assessment dimensions. Our study stands out as a pioneering effort in integrating an interactive hybrid text messaging and web-based chatbot-interfaced monitoring system that provides real-time responses tailored to patients’ needs during their chemotherapy for GI malignancies. This innovative approach mirrors the patient-provider interaction, simulating a comprehensive review of systems and offering personalized feedback aligned with individualized side effects. Notably, while a similar system was developed in a study by Richards et al [23], which explored a symptom-monitoring and feedback system for upper GI cancer surgery, their evaluation primarily rested on qualitative analysis. In contrast, our study delves into comprehensive metrics, encompassing diverse aspects like quality of life and patient activation. The novel blend of technology and patient-centered care in RT-CAMSS holds the potential to reshape patient experiences and outcomes in managing chemotherapy side effects for GI cancers.

### Study Limitations

We acknowledge limitations in our study design and execution. The pilot nature of the study, reflected in the small sample size, may impact the generalizability of findings, as it may not fully capture patient diversity in demographics, socioeconomic status, and treatment settings. Biases inherent to a single-arm pilot design also influence outcome interpretation. While provider follow-up alerts were omitted for patient empowerment, the observed lower frequency of severe symptoms could stem from academic center preparation. Future directions involve electronic health record integration for improved information exchange.

Postintervention feedback revealed reduced usage due to reasons like avoiding cancer reminders and symptom-free status. The lack of a control group precluded efficacy validation, a goal for future randomized controlled trials. Additionally, an Analytic Hierarchy Process approach [24] was not used, suggesting room for further refinement in assessment methods.

Our chatbot’s structured approach limits its understanding of nuanced patient contexts due to the absence of National Laguage Processing (NLP) capabilities. In addition to offering accessibility, NLP’s omission restricts deeper interaction. Future research may explore integrating NLP to enhance responsiveness to diverse patient expressions.

### Conclusions

This study successfully demonstrated the feasibility and acceptability of implementing an interactive text messaging-based and chatbot-interfaced symptom management system for patients with GI cancer undergoing intravenous chemotherapy. Moreover, our findings underscore the significance of patient engagement with the system, as evidenced by improved PAM results and heightened proactivity in taking actions to enhance and maintain health. Moving forward, future investigations should delve into the broader impacts of such a system on patients’ chemotherapy journeys and related metrics while also assessing its influence on patient engagement and adherence across various delivery modalities (eg, MyChart, chatbot, or text messaging). Furthermore, to ensure optimal functionality, the full integration of ePRO with electronic health record and clinical information systems would grant clinicians access to real-time ePRO data, facilitating proactive medical follow-up care. Such comprehensive and seamless integration holds the potential to enable timely clinical interventions and enhance personalized treatment decision-making.

## Abbreviations

CTCAE: Common Terminology Criteria for Adverse Events
ePRO: electronic patient-reported outcome
GI: gastrointestinal
PAM: Patient Activation Measure
PRO CTCAE: Patient-Reported Outcomes version of the Common Terminology Criteria for Adverse Events
REDCap: Research Electronic Data Capture
RT-CAMSS: Real-Time Chemotherapy-Associated Side Effects Monitoring Supportive System
